# Insights From Transplant Professionals on the Use of Social Media: Implications and Responsibilities

**DOI:** 10.3389/ti.2021.10181

**Published:** 2022-02-03

**Authors:** Shaifali Sandal, Arvinder Soin, Frank J. M. F. Dor, Elmi Muller, Ala Ali, Allison Tong, Albert Chan, Dorry L. Segev, Macey Levan

**Affiliations:** ^1^ Division of Nephrology, Department of Medicine, McGill University Health Centre, Montreal, QC, Canada; ^2^ Institute of Liver Transplantation and Regenerative Medicine, Medanta-The Medicity, Gurugram, India; ^3^ Imperial College Renal and Transplant Centre, Hammersmith Hospital, Imperial College Healthcare NHS Trust, London, United Kingdom; ^4^ Department of Surgery and Cancer, Imperial College, London, United Kingdom; ^5^ Division of General Surgery, Department of Surgery, University of Cape Town, Cape Town, South Africa; ^6^ Nephrology and Renal Transplantation Centre, The Medical City, Baghdad, Iraq; ^7^ Sydney School of Public Health, University of Sydney, Sydney, NSW, Australia; ^8^ Division of Hepatobiliary and Pancreatic Surgery and Liver Transplantation, Queen Mary Hospital, The University of Hong Kong, Hong Kong, China; ^9^ Department of Surgery, Division of Transplantation, Johns Hopkins University School of Medicine, Baltimore, MD, United States

**Keywords:** social media, transplantation, transplant professionals, advocacy, education

## Introduction

Social media (SoMe) is now widely accepted and used in medicine and considered a measure of scholarly output to support academic advancement at some institutions (1–4). In the field of transplantation, SoMe platforms, such as Twitter and Facebook, have been leveraged to promote both living and deceased donation, and for public engagement and outreach (5–11). These platforms are an important source of information on transplantation in many low and middle-income countries (12). Thus, academic institutions, and transplant organizations and journals are increasingly using SoMe to increase their visibility and footprint in the public domain and engage with the transplant community.

Despite this, there is still some hesitation among the transplant community to engage in SoMe. The majority of transplant surgeons in the United States perceive SoMe to be influential in increasing awareness about deceased organ donation and increasing living donation, yet 39% reported no SoMe outreach by their center (7). In Europe, while many transplant professionals reported using SoMe for work-related information, the majority do not engage in transplant-related campaigns (13). Thus, we reached out to eight leaders in transplantation who are known in their respective regions for their SoMe engagement or outreach or their work in the field of SoMe in transplantation. We specifically asked questions related to the opportunities and challenges of SoMe use related to their expertise which are presented below; some of the more general comments are concisely summarized in Table 1. For the purposes of this paper we focused on two platforms; one with the widest global reach (Facebook) and one most commonly used to disseminate knowledge (Twitter).

**TABLE 1:** Suggestions on social media (SoMe) use by transplant professionals.

**For transplant professionals**
Always
• Be professional
• Protect patient confidentiality
• Separate SoMe platforms into personal and professional use
• Carefully review the privacy settings of SoMe platforms to customize the content
• Share content from reputable sources only
• Be upfront about any conflicts of interest
• Be vigilant of transplant tourism and commercialization
• Abide by national laws and institutional policies
Consider
• Training in how to use SoMe platforms
• Sharing content with positive and genuine messages on organ donation and transplantation
• Tackling misinformation about transplantation
• Only sharing politically neutral content
• Engaging with patients but only with an appropriate oversight or ethical approval
Avoid
• Engaging in inflammatory content
• Using SoMe as a medium for personal attacks
• Sharing confidential and sensitive information that infringes on the intellectual property rights of others
**For transplant societies and organizations**
• Assist and empower transplant professionals in
o How to use SoMe to promote the interest of transplantation
o Maintain a SoMe presence
o Engage with the transplant community including patients and donors
• Explore the short- and long-term repercussions of the digital footprint of transplant professionals
• Explore avenues to assist professionals in tackling cyberbullying and harassment

### The Prolific and Enthusiastic Users

*Dr. Arvinder Soin*, a Transplant Surgeon from India, is considered to be a key medical influencer in various public health domains (most notably organ donation), and a leading healthcare figure on Twitter with over 97,000 followers. *Dr. Frank Dor*, a Transplant Surgeon from the United Kingdom is the current social media ambassador for the European Society of Organ Transplantation and set up the social media editorship of the Transplantation journal.

SS: Please share your overall thoughts on SoMe use by transplant professionals and the opportunities and responsibilities.

AS: SoMe platforms are excellent channels of communication that “democratize” the sharing of research, insights, and innovation; a process that has traditionally been confined to publications and conferences. Hashtags, such as #LiverTwitter, can inform and engage, and help glean breakthroughs. While SoMe engagement is a personal choice, lack of engagement by professionals leads to missed opportunities in expanding the field.

FD: SoMe is an underutilised instrument in professional communication, especially in the modern era of proper patient engagement. I am an enthusiastic SoMe user but establish boundaries across these platforms into personal and professional use. Professionals should engage in SoMe for patient/general public education, raising awareness about organ donation and transplantation, sharing scientific publications, promoting educational opportunities, such as courses and congresses, and influencing societal discussions (13).

SS: Dr. Soin, as a transplant figure with quite possibly the highest Twitter following of anyone in the field, what type of content do you create/share and any personal anecdotes you would like to mention?

AS: After establishing credibility in the field, I found it easier to expand my repertoire and engage on many medical issues, such as to keep the Indian public abreast on COVID-19. Also, I regularly share content with positive and genuine messages on organ donation and other aspects of transplantation. I make every attempt to be factually accurate and balanced when doing this, as the public accepts my transplantation-related content at face value. Also, I recommend avoiding taking a paternalistic stand to assert the superiority of one’s expertise; this may be counter-productive, or, at best, give only a short-term yield.

SS: Dr. Dor, as a SoMe ambassador of a leading organization what would you advise those who might be nervous about using SoMe?

FD: I believe that it is our professional duty to advocate for the best options for our patients and SoMe provides us with platforms to do so. However, I advocate for training, as the use of these platforms requires knowledge about how they work and what their potential pitfalls are. SoMe is a powerful tool, but as with all powerful tools, one needs to know how to use them safely and correctly to fit the purpose.

### The Prolific but Cautious Users

*Dr. Elmi Muller* is a prolific figure and Transplant Surgeon from South Africa whose work and profile has been featured in prominent scientific journals. She is involved in many organ transplant-related outreach and education programmes for the public. *Dr. Ala Ali*, a Transplant Nephrologist from Iraq, is an engaged professional and emerging transplant leader from the Middle East.

SS: Based on the comments made above, can you highlight some of the risks of SoMe use by transplant professionals?

EM: While I agree with what has been said, I recommend using SoMe cautiously as the message of a tweet or a story on Facebook, can fragmentize over time, be taken out of context, and create an ever-lasting digital footprint that can impact one’s personal and professional life years down the lane. There are many examples of individuals who applied for jobs and their SoMe profiles were scrutinized. In addition, there can be legal ramifications of sharing political views, making political statements, and violating patient confidentiality.

AA: We should consider shouldering responsibilities to advance the field of transplantation ethically and righteously and to help our patients as suggested by Drs. Dor and Soin. SoMe is being used to spread misinformation and as potential channels for transplant tourism and commercialization (14). For professionals there are several opportunities to deliver high-quality information to counter some of this misinformation but professional, ethical, and legal complexities exist in the developing world; the pandemic exaggerated these complexities.

SS: How do you recommend we address these emerging issues?

EM: Professionals need more training and guidance on how to use SoMe to our benefit and how to safely use it. There are opportunities for professional development, but how much it helps is not known. For example, it is very easy to re-tweet a paper and comment on its findings, but the repercussions of this in the short- and long-term need more exploration.

AA: I absolutely agree; we need more guidance that is tailored to the local context of practice and policy. While avoiding direct communication with patients, I recommend engaging in a medium, such as a patient’s education group, that permits professional responses only.

### The Transplant Researchers

*Dr. Allison Tong* is a Transplant Researcher from Australia and *Dr. Albert Chan* is a Transplant Surgeon from Hong Kong. Both are known for using SoMe for promoting their research endeavors and are well cited for their research contributions. Dr. Tong has over 24,000 citations of her scientific work with an h-index of 60 and Dr. Chan has over 4,500 citations of his scientific work with an h-index of 36.

SS: Can you share your thoughts on the use of SoMe for research in transplantation?

AT: SoMe is an important platform for sharing scientific information with opportunities for collaborations, exchanging opinions, and gaining different insights and perspectives. The impact on research findings has been demonstrated; a tweeted article was three times more likely to be downloaded compared with those that were not tweeted (2). SoMe is a great platform for patient engagement, especially for research conducted under the oversight of Institutional Review Boards.

AC: I agree. Given the transformation in information technology, SoMe use is inevitable and it can play some part in the promotion of transplantation and finding living donors. SoMe is useful in knowledge dissemination of novel surgical techniques, and an effective way to allow knowledge exchange and communication among professionals, or between professionals and the community.

SS: What are some of the risks that you have experienced?

AT: There can be a risk of oversimplifying or sensationalizing findings of a study or the “science,” which is open to public scrutiny. There is a need to ensure that one is making informed commentary and I caution against sharing confidential and sensitive information that infringes the intellectual property rights of others.

AC: I would add that there is a lack of verification mechanism, which substantially increases the risk of misinformation. Knowledge dissemination should be substantiated by publications in peer-reviewed journals with the link to citations in the postings or next to a “hashtag. ” Also, I recommend SoMe engagement to be politically neutral and restricted to purely scientific comments based on factual findings.

### The Political Advocates

*Dr. Dorry Segev* is a Transplant Surgeon from the United States who is a prominent transplant figure, researcher and globally known for his expertise in transplantation. His research has informed congressional bills and the HIV Organ Policy Equity Act that was signed into law. *Dr. Macey Levan* is a lawyer and living kidney donor recognized for her advocacy and ethics in living donation. Both have published extensively on SoMe use in transplantation.

SS: As someone who has been quite vocal on SoMe platforms, can you share your positive and negative experiences?

DS: SoMe engagement is rewarding professionally and personally that carries a risk of public scrutiny and occasional criticism and negative comments. Despite this, we should not silence professionals as these platforms can create dialogue and movements that can positively impact medicine and transplantation. I strongly recommend that professionals advocate for causes relevant to transplantation, including political conversations as it has significantly, sometimes negatively, intersected with the care of our patients, such as masking during the pandemic. There are opportunities to tackle misinformation and perpetuate new research and science.

ML: I find that SoMe can be seen as both disruptive and opportunistic as it allows quick and active communication but can also create conversations that can be superficial and passive. The American public craves information and access to it through social media channels, but attention spans are very short, with adults typically being able to pay attention to one task for 8 s.

SS: Dr. Henderson-Levan, as an ethicist can you provide any unique comments for SoMe use by transplant professionals?

ML: Organs from deceased donors are considered to be a national resource, and we should encourage transplant professionals to elevate organ donation and transplantation as part of an elevated public health conversation. We need to meet people where they are as we are in a public field that relies on the public to make it work, so we need to communicate with people in a quick and impactful way. Social media channels are great examples of ways to do this.

SS: Dr. Segev, any quick shot way to stay out of trouble when engaging on SoMe?

DS: First, set boundaries on SoMe platforms use into personal and professional. Second, pause before posting using the “front page of the New York times” litmus test. If the content and the message were to appear on the front page of any important newspaper, it must be acceptable to one’s personal and professional image.

### Implications of SoMe Use by Transplant Professionals for Patients

SS: With respect to SoMe use by transplant professionals, please share your thoughts on the implications to patients?

AS: SoMe conversations have the potential to change or garner public opinions that can perpetuate or debunk myths and fuel mass movements. I have had excellent engagement with sharing uplifting patient stories via SoMe (with informed consent of course), thus opening up avenues to apprise the masses about the benefits of organ donation and transplantation. I believe this has helped many of my patients and acknowledged the contributions of living donors.

FD: It is important that professionals engage in SoMe for patient/general public education and raising awareness about organ donation and transplantation. SoMe can reach a lot of people that professionals can’t reach normally in such magnitude. It is important to create and enlarge networks to increase the impact on our field. With proper training and guidance, I strongly believe our patients will benefit from transplant professional engagement.

EM: Transplant professionals are always exposed and always expected to maintain the highest level of ethics and professionalism. Comments can be taken out of context, and there may be legal ramifications. There are risks of violating patient confidentiality. I recommend transplant leadership considers exploring the risks and benefits to patients, donors and the public. This will better help assess how to engage in SoMe that benefit our patients and how to minimize the associated risks.

AA: SoMe provides opportunities for transplant professionals to deliver high-quality information to patients. I would consider engaging in a medium, such as a patient’s education group, that permits professional responses only. One should consider shouldering responsibilities to advance the field of transplantation in an ethical and righteous manner, and this can be of direct and indirect benefits to patients. In addition, the Declaration of Istanbul may consider updating its preamble surrounding SoMe use by professionals.

AT: SoMe can be useful for connecting with and engaging patients or other stakeholders in research; research benefits patients and improves their experiences, outcomes and health. However, there are risks. For recruitment, one should always go through approval by an Institutional Review Board to ensure messages are appropriate (i.e., not coercive). As long as we refrain from giving personal medical advice, promoting treatments, asking for personal medical history/information and similar unethical practices, I do think our patients will benefit from SoMe engagement by transplant researchers.

AC: SoMe is helpful to patients and to increasing organ donation. Maintaining patient confidentiality is the main risk to patients.

DS: I recommend setting boundaries on SoMe usage into personal and professional use. My patients follow me on Twitter but I never engage with them directly and instead will have a private conversation with them. I share my updated research outputs, ongoing studies and other exciting news. I have had a tremendous response to my work on COVID-19 vaccination to transplant patients. This has been of direct benefit to patients, especially in the midst of a pandemic.

ML: It is common now to use SoMe as a tool for research participation. Meeting these participants where they are, provides us insight into patient-oriented questions, and helps them feel the benefit and purpose from their participation. However, it is important to be mindful of what patients perceive from your SoMe persona while still ensuring you are able to hold your own professional and personal boundaries. When communicating with patients, it also reflects on associated institutions. In the Twitter era, if a patient does not like an interaction in a care facility, they can share these opinions both with the institution and with the public.

## Conclusion

Our collective expertise suggests that a lack of SoMe engagement leads to several missed opportunities in advancing the interests of our patients, our field, and our careers. We believe transplant professionals should consider maintaining a SoMe presence, engaging with the transplant community, and debating hot topics while seeking guidance on how to do so safely and effectively. We have summarized our collective suggestions in Table 1.

## Abbreviations

SoMe, social media.
